# Neurodevelopment at 2 years corrected age among Vietnamese preterm infants

**DOI:** 10.1136/archdischild-2019-316967

**Published:** 2019-07-12

**Authors:** Chuong Huu Thieu Do, Alexandra Yasmin Kruse, Bridget Wills, Saraswathy Sabanathan, Hannah Clapham, Freddy Karup Pedersen, Thanh Ngoc Pham, Phuc Minh Vu, Malene Landbo Børresen

**Affiliations:** 1 Neonatal Intensive Care Unit, Children’s Hospital 1, Ho Chi Minh City, Vietnam; 2 Global Health Unit, Department of Paediatric and Adolescence, Copenhagen University Hospital - Rigshospitalet, Copenhagen, Denmark; 3 Oxford University Clinical Research Unit, Hospital for Tropical Diseases, Ho Chi Minh City, Vietnam; 4 Centre for Tropical Medicine and Global Health, Nuffield Department of Medicine, University of Oxford, Oxford, UK; 5 Department of Pediatrics, University of Medicine and Pharmacy at Ho Chi Minh City, Ho Chi Minh City, Vietnam

**Keywords:** preterm infant, intensive care, neurodevelopment, Bayley scale, low resource setting, middle-income country

## Abstract

**Background:**

Preterm infants are at risk of neurodevelopmental delay, but data on long-term outcomes in low-income and middle-income countries remain scarce.

**Objectives:**

To examine neurodevelopment using Bayley Scales of Infant and Toddler Development-3rd edition (Bayley-III) and neurological findings in 2-year-old preterm infants, and to compare with healthy Vietnamese infants. Further, to assess factors associated with neurodevelopmental impairment.

**Design and setting:**

Cohort study to follow up preterm infants discharged from a neonatal intensive care unit (NICU) of a tertiary children’s hospital in Vietnam.

**Participants:**

Infants born at <37 weeks of gestational age.

**Main outcomes:**

Bayley-III assessment and neurological examination at 2-year corrected age (CA) compared with healthy Vietnamese infants.

**Results:**

Of 294 NICU preterm infants, Bayley-III scores of all 184/243 (76%) survivors at 2 years CA were significantly lower than those of healthy Vietnamese peers in all three domains: cognition (mean (SD): 84.5 (8.6) vs 91.4 (7.5), p<0.001), language (mean (SD): 88.7 (12.5) vs 95.9 (11.9), p<0.001) and motor (mean (SD): 93.1 (9.0) vs 96.8 (9.3), p=0.003). The mean differences in Bayley-III scores between preterm and healthy Vietnamese infants were −6.9 (−9.1 to −4.7), −7.2 (−10.5 to −3.8) and −3.7 (−6.1 to −1.2) for cognitive, language and motor scores, respectively. The prevalence of neurodevelopmental impairment was 17% for cognitive, 8% for language and 4% for motor performance. In total, 7% were diagnosed with cerebral palsy. Higher maternal education was positively associated with infant neurodevelopment (OR 0.32, 95% CI 0.11 to 0.94).

**Conclusions:**

Vietnamese preterm infants in need of neonatal intensive care showed poor neurodevelopment at 2 years. Higher maternal education was positively associated with infant neurodevelopment. Standard follow-up programmes for preterm infants should be considered in low-resource settings.

**What is already known on this topic?**

- Preterm infants are at high risk of neurodevelopmental impairment.
- Data on long-term outcomes for preterm infants are scarce in low-income and middle-income countries.
- Both biological and environmental factors influence child development.

**What this study adds?**

- Vietnamese preterm infants in need of neonatal intensive care have lower neurodevelopmental scores compared with healthy Vietnamese peers.
- Higher maternal education is positively associated with infant neurodevelopment in limited-resource settings.
- To identify preterm infants with neurodevelopmental impairment, follow-up programmes should be considered in Vietnam and other low-income and middle-income countries.

## Introduction

Premature birth, defined as birth before 37 completed weeks of gestation, accounts for approximately 10% of live births worldwide^1^ and is one of the leading causes of death among children under the age of 5 years.^2^ In Vietnam, the rate of premature birth is 94/1000 live births,^3^ which is similar to that of other low-income and middle-income countries (LMICs) in Southeast Asia, though Vietnam has a lower rate of neonatal death from prematurity (4.3 vs 8.8/1000 live births in the region).^2^ With progressive improvements in neonatal care in the country, an increasing number of very preterm infants are now surviving the perinatal period. However, the gains in survival have concomitantly raised the question of long-term outcomes for these infants.

Challenges in the care of preterm infants are reflected in high mortality and also in noticeable morbidity.^4 5^ Infants born prematurely are at high risk of injury, particularly to the immature brain, which may be critical for their development with possible adverse effects lasting into adulthood.^6^ Consequently, neurodevelopmental impairments, including cognitive, language, motor and neurosensory impairments as well as behavioural disorders, occur more frequently in this vulnerable group.^7^ Among preterm infants, there is an even higher risk of impairment among extremely and very preterm (EVP) infants (gestational age: GA <32 weeks) compared with their moderate and late preterm (MLP) counterparts (GA 32–36 weeks).^7 8^ Despite accumulating evidence in the literature, data from Vietnam and other LMICs are limited due to a lack of follow-up programmes for high-risk infants in general and for preterm infants in particular.^9^

One of the main difficulties in establishing a well-organised follow-up programme has been the absence of neurodevelopmental assessment tools validated in a similar context. The Bayley Scales of Infant and Toddler Development-3rd edition (Bayley-III) is one of the most frequently used tools to evaluate neurodevelopmental delay, with the tests covering cognitive, language and motor skills considered highly informative in terms of delineating outcomes for preterm infants.^10 11^ Recently, the Bayley-III was adapted and validated for use in Vietnam, and is now being implemented as a research tool in several local follow-up programmes.

Information on long-term outcomes is crucial for both policy making and future planning for healthcare, social and educational services and for counselling caregivers about expected outcomes following preterm birth.^12^ To our knowledge, long-term data for Vietnamese preterm infants have not been published. We hypothesised that the Bayley-III scores of preterm Vietnamese infants were different from those of healthy Vietnamese infants. Therefore, we examined neurodevelopmental outcomes in a hospital-based cohort of preterm infants discharged from a neonatal intensive care unit (NICU), using the Bayley-III at 2-year corrected age (CA). We also estimated the prevalence of abnormal neurological examination and attempted to assess risk factors for poor outcome.

## Methods

Our study was conducted at the NICU at Children’s Hospital 1 (CH1) in Ho Chi Minh City, one of the two major tertiary centres responsible for critical care of newborns in southern Vietnam. The NICU has 30 beds and 1200 admissions annually, of which the vast majority are transferred from healthcare facilities providing obstetric care and the others are admitted from home via the emergency room. The study was approved by the Institutional Review Board of CH1 and written parental consent was obtained for each study participant.

All preterm newborns admitted to the unit from July 2013 to September 2014 were eligible for enrolment if they fulfilled the following criteria: <37 completed weeks gestation at birth, age at admission <29 days, no congenital brain malformations or chromosomal anomalies. Newborns admitted to NICU for <72 hours for stabilisation only, or for treatment of retinopathy of prematurity (ROP), were excluded.

After discharge, participating infants were followed up until 24-month CA.^13^ Demographic data were obtained at the first visit including living place, primary caregiver, maternal factors (age, educational level and occupation) and ethnicity. Subsequently, the hospital files were reviewed to obtain information on a range of clinical characteristics (Appendix). Cerebral ultrasound was conducted either during the hospital stay or before 6-month CA and the worst result for each infant is reported here. Major birth defects were identified using the Manual of Operations of the Vermont Oxford Network (http://public.vtoxford.org). Hearing and visual function were assessed by caregivers’ report and neurological examination, and hearing screening using otoacoustic emission testing was carried out before discharge or at the first visit before 6 months. If there was any suspicion of impairment, the infant was referred to an otolaryngologist for auditory brainstem responses, or an ophthalmologist for formal assessment of vision, as appropriate.

At the 24-month CA visit, neurodevelopment was assessed using a locally adapted Bayley-III assessment tool, previously shown to have acceptable reliability and validity in a Vietnamese population. All tests were conducted by a group of six certified Bayley assessors who were blind to the details of the infants’ birth and hospital course. Tests were performed at CH1 in a quiet, air-conditioned room, and the results were recorded on standard assessment forms, licensed by Pearson publisher.^10^ The Bayley-III scores obtained from a cohort of healthy Vietnamese infants were used as a reference for comparison and classification. All infants were also examined by the principal investigator using the method of Amiel-Tison’s neurological examination.^14^ Infants who scored 2 on any neurological finding at 2-year CA were considered to have a moderate/severe abnormality and were referred to a neurologist for confirmation. Cerebral palsy (CP) was diagnosed according to the European guidelines^15^ and categorised using the Gross Motor Function Classification System (GMFCS) ranging from levels 1 to 5, in which a higher level indicates more severe motor impairment.^16^

We classified overall outcome using a composite of the Bayley-III scores, the neurological examination and the sensory function assessment. Infants with missing information for one or more of these assessments that could have altered their final category were considered unclassifiable. The category ‘significant neurodevelopmental impairment (significant NDI)’ included infants with a Bayley-III score on any composite score <−2SD, a GMFCS level 3–5, moderate/severe neurological abnormality, or blindness or deafness. The category ‘any neurodevelopmental impairment (any NDI)’ included infants with a Bayley-III score on any composite score <−1SD, a GMFCS level ≥1 or any neurological abnormality.^17^ This category encompasses all infants in the significant NDI group.

### Statistical analysis

Summary statistics are presented as mean (SD) or median (IQR) for continuous variables, and in absolute counts and percentages for categorical variables. Bayley-III scores between preterm Vietnamese infants and healthy Vietnamese infants were compared using Student’s t-test and presented with mean difference (95% CI). Effect sizes were also presented using Cohen’s d method defined by the difference between two means divided by the pooled SD of those means.^18^ Regarding sample size, our approach was pragmatic as the Bayley-III scores of preterm infants at 2 years have not been reported in Vietnam. However, with a sample size of 184 participants, the study was powered to detect a difference of 4.4 points for composite scores and 0.9 points for scale scores with 80.0% power based on a two-sided test with type I error of 5.0%. A difference of ≥5 points for composite scores and ≥1 point for scale score was considered clinical importance.^19^

For each Bayley-III domain, the Z-score was calculated by comparing the infant’s score with the mean and SD of the score for the same domain derived from healthy Vietnamese infants. The reference population comprised a group of 78 full-term healthy Vietnamese infants assessed at 24 months. These infants had been recruited for the validation of the Bayley-III adaptation and as the control group for another study on hand-foot-and-mouth disease (see online supplementary appendix). These healthy infants had scores lower than the standardised US reference scores of 100 (SD=15).

10.1136/archdischild-2019-316967.supp1 Supplementary data

Using univariable and multivariable logistic regression analyses, we assessed potential associations between the main outcome of interest, ‘any NDI’ and the following variables— gender,^20^GA,^21^ multiple birth,^22^ maternal age, maternal education,^23^ need for mechanical ventilation,^24^ occurrence of sepsis,^25^ development of chronic lung disease^17^ and need for surgery.^26^ These candidate covariates were identified by a review of the literature on risk factors for poor outcome and formed our initial full conceptual model (see online supplementary appendix). All statistical analyses were performed using R software V.3.5.0 and the level of statistical significance was set at p<0.05.

## Results

**Figure 1:**
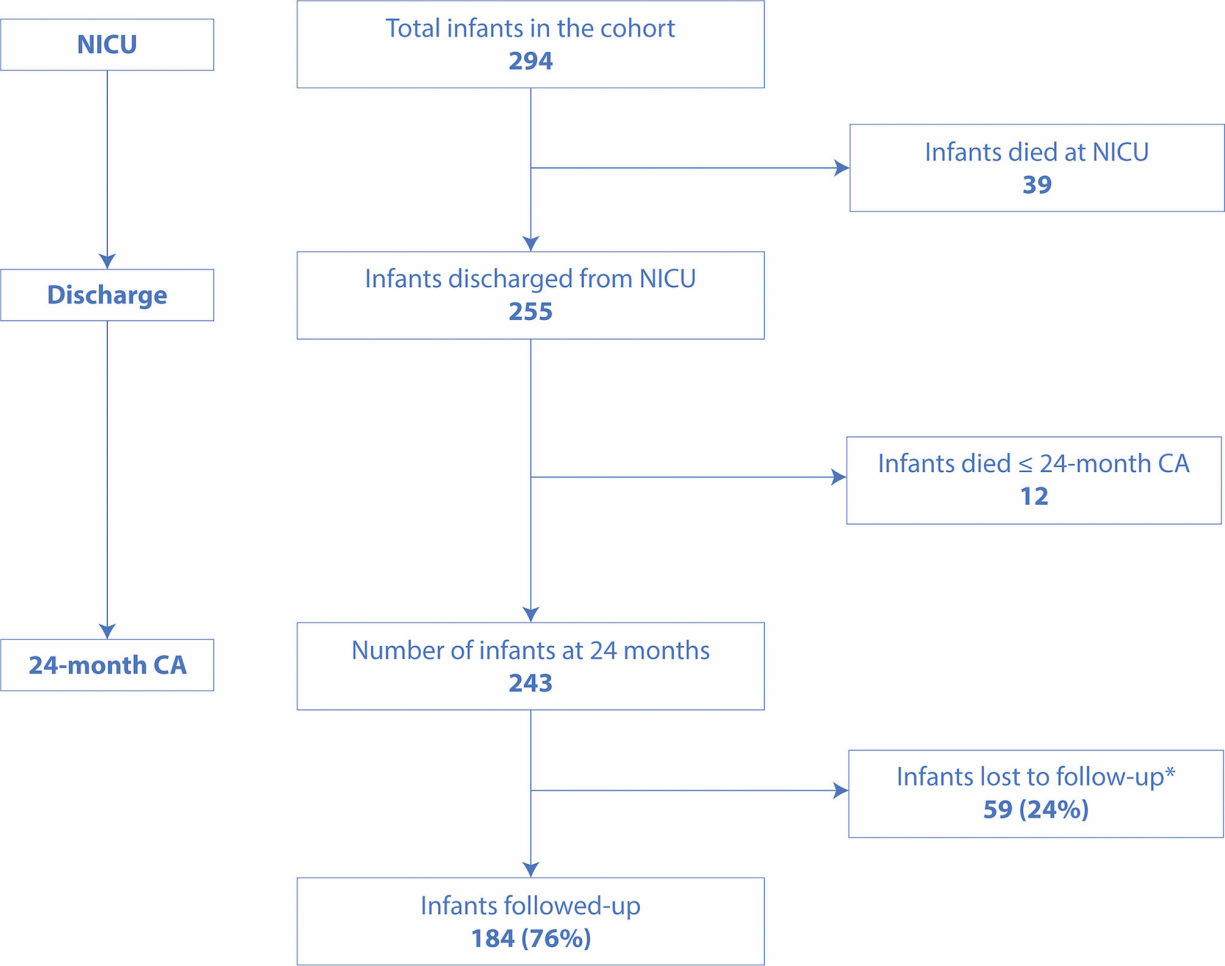
Flow chart of preterm infants from NICU discharge to 24-month neurodevelopmental assessment. NICU, neonatal intensive care unit. CA, corrected age. *Reasons for attrition (number of infants): being unable to contact (25), travel problems (11), language of minor ethnics (3), living abroad (3), well-being child (5) and unspecified (12).

Of the 294 preterm infants (all out-born) enrolled during the study period, 243 survived to 2 years CA, and of these 184 (76%) were evaluated at this time (figure 1). Among these infants, GA ranged from 26 completed weeks to 36 completed weeks. The mean (SD) birth weight (BW) and mean (SD) GA of followed-up infants were 1754 (484) g and 31.6 (2.5) weeks compared with 1866 (438) g and 32.3 (2.2) weeks of those who were lost to follow-up.

### Demographic characteristics

Table 1 presents the demographic characteristics for the two GA groups (EVP vs MLP infants); these characteristics were similar between the two GA groups, except for BW and GA by design.

**Table 1:** Demographic characteristics of preterm infants assessed at 2 years corrected age

Characteristics	Number (%) or mean (SD)		
	Gestational age (GA)		
	GA <32 weeks (n=86)	GA ≥32 weeks (n=98)	Total (n=184)
Boys, n (%)	52 (60)	65 (66)	117 (64)
Birth weight (g), mean (SD)	1377 (251)	2085 (387)	1754 (484)
Gestational age (week), mean (SD)	29.4 (1.5)	33.5 (1.4)	31.6 (2.5)
GA distribution, n (%)			
<28 weeks	12 (14)	_	12 (7)
28 weeks–<32 weeks	74 (86)	_	74 (40)
32 weeks–<34 weeks	_	53 (54)	53 (29)
34 weeks–<37 weeks	_	45 (46)	45 (24)
Multiple births (all twins), n (%)	12 (14)	14 (14)	26 (14)
Living in the city of study-site, n (%)	13 (15)	27 (28)	40 (22)
Mother age at birth (years), mean (SD)	28.6 (5.9)	29.0 (6.2)	28.8 (6.0)
Maternal education*, n (%)			
Elementary school or less	25 (29)	33 (34)	58 (32)
High school (junior or senior)	52 (60)	52 (53)	104 (57)
College or higher level	9 (10)	13 (13)	22 (12)
Maternal occupation, n (%)			
Skilled job†	16 (19)	18 (18)	34 (18)
Housewife	28 (33)	24 (24)	52 (28)
Farmer	12 (14)	8 (8)	20 (11)
Others‡	30 (35)	48 (49)	78 (42)
Primary care by parents§, n (%)	78 (90)	91 (93)	169 (92)
Ethnic minority (non-Kinh)¶, n (%)	1 (1)	2 (2)	3 (2)

Of note, data on income status were not available due to indeterminate responses from parents’ perspective.

Because of rounding, percentages may not total 100.

*Educational system consists of the basic education and the higher education. Twelve-year basic education includes 5 years in elementary school, 4 years in junior high school and 3 years in senior high school. The higher education includes college, undergraduate and postgraduate education in the universities.

†Skilled job refers to professional and intellectual work.

‡Other jobs refer to unskilled labour and shopkeeper.

§Other primary caregivers were grandparents or other relatives.

¶Ethnic minority includes Khmer, Chinese.

Boys accounted for 117/184 (64%) of all the infants followed up, but the mean (SD) of BW and GA were similar between boys and girls (BW: boys, 1788 (444) g vs girls, 1695 (546) g, GA: boys, 31.7 (2.4) weeks vs girls, 31.6 (2.7) weeks).

### Clinical course and treatment characteristics

Table 2 presents characteristics of the clinical course and treatment during the neonatal period for the two GA groups.

**Table 2:** Characteristics of clinical course and treatment in the Neonatal Intensive Care Unit

Characteristics	Number (%) or median (IQR)		
	Gestational age (GA)		
	GA <32 weeks (n=86)	GA ≥32 weeks (n=98)	Total (n=184)
5 min Apgar score ≤6, n (%)	6 (7)	0 (0)	6 (3)
Major birth defects*, n (%)	4 (5)	21 (21)	25 (14)
Mechanical ventilation, n (%)	44 (51)	48 (49)	92 (50)
Chronic lung disease, n (%)	28 (33)	6 (6)	34 (18)
With postnatal corticosteroids	6 (21)	2 (33)	8 (24)
Without postnatal corticosteroids	22 (79)	4 (67)	26 (76)
Sepsis, n (%)	67 (78)	56 (57)	123 (67)
Positive blood culture	8 (12)	14 (25)	22 (18)
Negative blood culture†	59 (88)	42 (75)	101 (82)
Shock‡ during hospital stay, n (%)	11 (13)	15 (15)	26 (14)
CPR during hospital stay, n (%)	7 (8)	5 (5)	12 (7)
Necrotising enterocolitis, n (%)	8 (9)	3 (3)	11 (6)
Surgery§, n (%)	12 (14)	21 (21)	33 (18)
Clinical seizure, n (%)	0 (0)	3 (3)	3 (2)
Abnormal cerebral ultrasound, n (%)	19/86 (22)	18/91 (20)	37/177 (21)
Periventricular leukomalacia	1 (5)	1 (6)	2 (5)
Intracranial haemorrhage grade I or II	14 (74)	12 (67)	26 (70)
Intracranial haemorrhage grade III or IV	2 (11)	1 (6)	3 (8)
Other¶	2 (11)	4 (22)	6 (16)
Laser ROP, n (%)	12/82 (15)	1/49 (2)	13/131 (10)
Length of stay (days), median (IQR)	48 (36, 71)	24 (15, 35)	34 (21, 51)

Antenatal steroid use was not reported because of insufficient information from referral letters between hospitals and only indicated for impending premature delivery with GA <35 weeks.

There were two infants whose data could not be completed because of missing information on the medical records.

Because of rounding, percentages may not total 100.

*Major birth defects included esophageal atresia (5), intestinal atresia (4), imperforate anus (3) gastroschisis (9), diaphragmatic hernia (2), sacrococcygeal teratoma (1) and pulmonary atresia (1).

†Suspected sepsis based on clinical signs and biomarkers for septicemia.

‡Shock is considered as circulatory failure that requires vasopressors and fluid resuscitation.

§Surgery includes repairs of congenital malformations (24), volvulus from intestinal malrotation (1), peritonitis due to gastrointestinal perforation (4) and patent ductus arteriosus ligation (4).

¶Other abnormalities on cerebral ultrasound include calcified nodes at the putamen (1), mild enlargement of frontal subarachnoid space (1), mild enlargement of posterior fossa (1), mild enlargement of anterior horn of left lateral ventricle (1), mild enlargement of both lateral ventricles (1) and mild hydrocephalus plus mildly enlarged posterior fossa (1).

CPR, cardiopulmonary resuscitation; ROP, retinopathy of prematurity.

In total, major birth defects were diagnosed in 25/184 (14%) infants; these defects were more frequent in MLP infants. In contrast, the EVP infants spent more days in the hospital and had a higher risk of acquiring chronic lung disease compared with MLP infants. Sepsis was diagnosed clinically in 67/86 (78%) infants in the EVP group compared with 56/98 (57%) in the MLP group. Among them, confirmed sepsis (at least one positive culture from blood or another sterile site) was only identified in 22/123 (18%) infants. As expected, ROP laser treatment was administered mostly in EVP infants, EVP versus MLP: 12/82 (15%) versus 1/49 (2%).

A total of 177 infants had at least one cerebral ultrasound performed either during the NICU stay (148/177, 84%) or after discharge (29/177, 16%). Among them, 140 (79%) infants had normal scans, while mild intraventricular haemorrhage (IVH) (grades 1 or 2) was identified in 26 (15%), severe IVH (grades 3 or 4) in 3 (2%) and periventricular leukomalacia (PVL) in 2 (1%) infants.

### Neurodevelopmental outcomes

The mean (SD) for age at Bayley-III assessment was 23.9 (0.6) months CA, with a range from 22 months 29 days to 25 months 15 days CA (183/184), except for one infant assessed at 20 months 24 days. Table 3 presents the Bayley-III scores of preterm infants compared with healthy Vietnamese infants.

**Table 3:** Neurodevelopmental outcomes at 2 years corrected age of preterm Vietnamese infants compared with healthy Vietnamese infants

Neurodevelopmental domains		Preterm Vietnamese infants (n=184)	Healthy Vietnamese infants* (n=78)	Mean difference (95% CI)	P value†	Effect size Cohen’s d
Cognitive composite score	Mean (SD)	84.5 (8.6)	91.4 (7.5)	−6.9 (−9.1 to −4.7)	<0.001	0.83
(n=184)	Z-score, mean (SD)‡	−0.91 (1.13)	–			
	<−2 SDs, n (%)	32/184 (17%)	–			
Language composite score	Mean (SD)	88.7 (12.5)	95.9 (11.9)	−7.2 (−10.5 to −3.8)	<0.001	0.58
(n=179)	Z-score, mean (SD)	−0.60 (1.05)	–			
	<−2 SDs, n (%)	14/179 (8%)	–			
Motor composite score	Mean (SD)	93.1 (9.0)	96.8 (9.3)	−3.7 (−6.1 to −1.2)	0.003	0.40
(n=180)	Z-score, mean (SD)	−0.39 (0.96)	–			
	<−2 SDs, n (%)	8/180 (4%)	–			
Cognitive subtest	Mean (SD)	6.9 (1.7)	8.3 (1.5)	−1.4 (−1.8 to −0.9)	<0.001	0.83
(n=184)	Z-score, mean (SD)	0.91 (1.13)	–			
	<−2 SDs, n (%)	32/184 (17%)	–			
Receptive language subtest	Mean (SD)	7.7 (2.0)	8.9 (2.1)	−1.2 (−1.7 to −0.6)	<0.001	0.57
(n=179)	Z-score, mean (SD)	−0.56 (0.78)	–			
	<−2 SDs, n (%)	13/179 (7%)	–			
Expressive language subtest	Mean (SD)	8.3 (2.7)	9.6 (2.1)	−1.3 (−2.0 to −0.6)	<0.001	0.50
(n=179)	Z-score, mean (SD)	−0.55 (1.13)	–			
	<−2 SDs, n (%)	11/179 (6%)	–			
Fine motor subtest	Mean (SD)	8.6 (1.9)	9.6 (1.8)	−1.1 (−1.6 to −0.6)	<0.001	0.56
(n=183)	Z-score, mean (SD)	−0.60 (1.10)	–			
	<−2 SDs, n (%)	19/183 (10%)	–			
Gross motor subtest	Mean (SD)	9.0 (1.9)	9.2 (2.1)	−0.2 (−0.8 to 0.3)	0.37	0.12
(n=180)	Z-score, mean (SD)	−0.11 (0.86)	–			
	<−2 SDs, n (%)	3/180 (2%)	–			

The Bayley-III generates a raw score that is converted to a scale score and then combined to yield a composite score. It consists of five subscale scores: cognitive, receptive language, expressive language, fine motor and gross motor and three composite scores: cognitive, language and motor composite scores, with the lower scores indicating a greater degree of developmental delay.

*Healthy Vietnamese infants were born full-term, without history of severe illness (cardiac, epilepsy and HIV), without intensive care admission, and without known developmental delay.

†Student’s t-test was used to compare Bayley-III scores between preterm and healthy Vietnamese infants.

‡Bayley-III Z-scores of preterm infants were calculated using mean and SD of Bayley-III scores of healthy Vietnamese infants.

The mean (SD) of all scale and composite Bayley-III scores for the preterm infants were significantly lower than those of the healthy Vietnamese infants, except for the gross motor score which was comparable (9.0 (1.9) vs 9.2 (2.1), p=0.37). The mean (SD) of the composite cognitive Z-score was −0.91 (1.13), whereas the mean (SD) of the composite language and composite motor Z-scores were −0.60 (1.05) and −0.39 (0.96). The mean differences in Bayley-III scores between preterm and healthy Vietnamese infants were −6.9 (−9.1 to −4.7), −7.2 (−10.5 to −3.8) and −3.7 (−6.1 to −1.2) for cognitive, language and motor scores, respectively. The prevalence of moderate/severe NDI was 32/184 (17%) for cognitive, 14/179 (8%) for language and 8/180 (4%) for motor performance. The percentages of infants classified as having mild (ie, scores from −2SD to <−1SD) or moderate/severe (ie, scores <−2SD) impairment in each score are presented in table A in the Appendix.

Neurological examination was abnormal in 15/181 (8%) infants, with CP diagnosed in 13/181 (7%) infants, including two (1%) unable to walk (GMFCS level 3) due to spastic diplegia and other 11 (6%) able to walk but with some restriction (GMFCS level 1 or 2). The other two infants had mild neurological findings, while three infants were uncooperative with the examination. Blindness and deafness were not detected in any infant in our cohort. A total of 101/184 (55%) infants met our definition for ‘any NDI’ at the 2-year follow-up, among whom 43/183 (23%) infants were classified as ‘significant NDI’. One infant who attended could not be classified for the significant NDI outcome.

**Figure 2:**
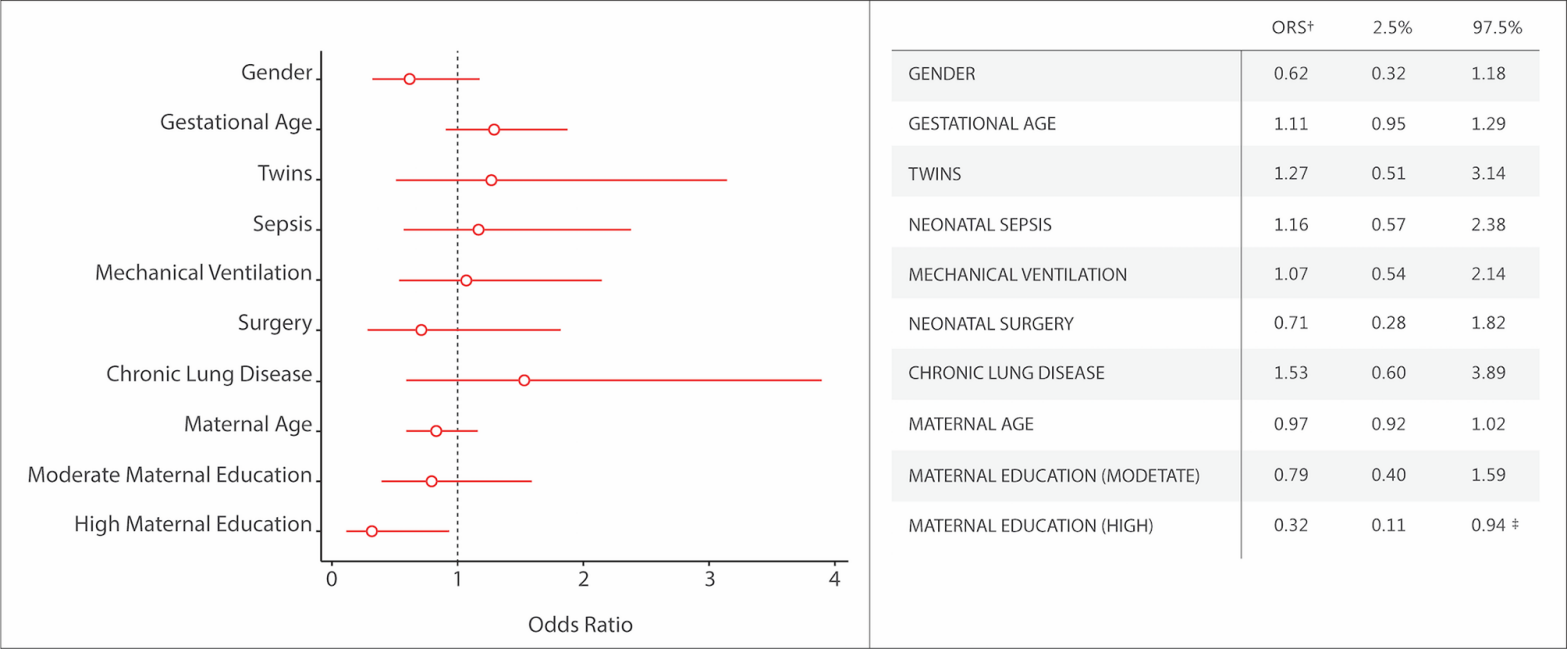
Adjusted risk factors for any neurodevelopmental impairment.* (OR and 95% CI). *Any neurodevelopmental impairment is defined as a Bayley-III score on any composite score <−1 SD or GMFCS level ≥1 or any neurological abnormality.^17^ †OR estimated as follows: for gender, girl relative to boy; for gestational age, increment in each week of gestation; for mechanical ventilation, at least 1 day treated with ventilator relative to non-ventilator; for neonatal surgery, at least one surgery relative to non-surgery; for maternal age, increment in each year of age; for maternal education, low maternal education used as reference for moderate and high maternal education. ‡P value <0.05.

Figure 2 depicts associations between demographic features and clinical characteristics during neonatal hospitalisation, and neurodevelopmental outcome, using ‘any NDI’ as the dependent variable. Among the risk factors included in the model, only maternal education at a college or higher level compared with low educational level (Appendix) was likely to reduce the odds of ‘any NDI’ in both unadjusted analysis (OR 0.28; 95% CI 0.10 to 0.81) and adjusted analysis (OR 0.32; 95% CI 0.11 to 0.94).

## Discussion

Our study reports neurodevelopmental outcomes for Vietnamese preterm infants at 2-year CA and contributes to the evidence of adverse outcomes for preterm infants in LMICs.^27^ We found that preterm infants had significantly lower neurodevelopmental scores compared with healthy Vietnamese counterparts in all three domains: cognition, language and motor. Among neurodevelopmental skills, cognitive scores were lowest, followed by language and motor scores, and these scores were lower than those reported by studies on extremely preterm infants in developed countries.^28^ Due to small sample size in each EVP and MLP groups, we cannot demonstrate the difference in Bayley-III scores between two GA groups, although several studies showed an inverse relationship between GA and NDIs.^21 29^ Interestingly, we found that higher maternal education was associated with favourable neurodevelopmental performance of preterm infants, similar to previous studies.^30^

The low cognitive and language scores of our preterm infants are consistent with previous reports,^7 29 31^but in addition to the vulnerability of preterm birth, these findings should be scrutinised in the light of Vietnamese culture.^32^ With low BW infants, Vietnamese caregivers tend to focus primarily on physical growth rather than on mental development. As well as lack of knowledge of techniques to maximise cognitive potential, the time spent on interactive play and the diversity of available toys have been found to be limited.^33 34^ This may explain the high percentages of participants with some impairment in cognition (42%) and language (31%).

Although the motor composite score was significantly lower than the norm, the gross motor scores were comparable. Our results are consistent with a meta-analysis indicating that preterm infants are typically in the normal range, but at the lower end of motor performance compared with full-term counterparts.^35^ The rate of CP in our cohort was 7% at 24-month CA, similar to other results,^36^ and two infants with GMFCS level 3 had manifest spastic diplegia, the most common form of CP in preterm infants.^37^ Of note, brain injuries detected by cerebral imaging including IVH and PVL have been demonstrated to be strong predictors for adverse motor outcomes, especially affecting gross motor function.^38^ However, these findings were identified in only 3% of our infants and this may partly explain the better gross motor performance.

### Public health applications

Our findings suggest that a formal follow-up programme similar to those used in high-income countries should be considered for preterm infants in LMICs, especially for those requiring neonatal intensive care. The study indicates that the anticipated poor neurodevelopment in these infants may be alleviated by involvement and education of their caregivers. Therefore, caregivers need to understand the risk of impairment and their role in maximising their child’s cognitive and language potential. A recent meta-analysis concluded that the impact of perinatal risk factors on the cognitive development of preterm infants is likely to lessen over time, whereas the effects of environmental factors become more prominent.^39^ Thus, in addition to applying up-to-date newborn care for preterm infants, development of effective systems for early detection and appropriate intervention for any ensuing problems should be considered. This first exploratory study of follow-up of preterm infants may provide a benchmark for future improvement in Vietnam and similar settings. Further studies on the benefit of early intervention and the active role of caregivers in improving long-term neurodevelopment for preterm infants is important, considering also the cultural context.

### Strengths and limitations

The prospective design is one of the strengths of our study and the cohort represents one of the largest and longest followed-up groups of preterm infants in Southeast Asia. Further, the Bayley-III assessments were conducted by independent examiners unaware of the children’s history, and the tool that we used had been previously adapted and validated for Vietnamese infants. On the other hand, we acknowledge several limitations including the bias inherent in a single-centre study involving the most severe out-born newborns and only representing a certain region of Vietnam. These issues may limit the generalisation of our findings to the broader population of children born prematurely across the country. Furthermore, nearly 25% of the infants were lost to follow-up, potentially resulting in bias. Finally, the low predictive ability of Bayley assessment in early childhood^5 40^  raises the need for longer follow-up into the school years and adolescent age.

## Conclusion

In conclusion, preterm Vietnamese infants in need of intensive neonatal care showed poor performance in all domains of neurodevelopment assessed at 2 years CA. Higher maternal education was positively associated with neurodevelopmental performance of these preterm infants. Our study suggests consideration of long-term neonatal follow-up programmes in LMICs that could assist in ensuring this vulnerable group of children fulfil their potential.
