# Supplementary material for: Neurodevelopment at 2 years corrected age among Vietnamese preterm infants
Source: Arch Dis Child. 2019 Jul 12;105(2):134–40. doi: 10.1136/archdischild-2019-316967 (PMC7025726; doi:10.1136/archdischild-2019-316967)
Supplement: Supplementary data [file archdischild-2019-316967supp001.pdf]

Appendix

1. Children's Hospital 1 does not provide obstetric/maternal care and the criteria for admission to NICU at Children's Hospital 1 are as follows:

- preterm newborns with BW less than 2000 gram or
- newborns in need of respiratory support or cardiovascular support.
- newborns in need of surgery in the neonatal period.

2. Gestational age

Gestational age (GA) was defined as completed weeks of gestation and was determined using one of the three following methods in order of priority: antenatal ultrasound before 20 weeks GA, the last menstrual day, or clinical examination applying the New Ballard Score.

3. Maternal characteristics

- ***Maternal education*** was classified as one of three grades: lower than high school, a high-school graduate, and college/higher level.
- ***Maternal occupation*** was classified as skilled job, housewife, farmer and unskilled labor.

4. Clinical characteristics

- ***Chronic lung disease*** (CLD) was diagnosed in infants with oxygen dependence persisting for the first 28 days from birth, plus a typical chest radiograph showing bronchopulmonary dysplasia (ill-defined reticular markings plus interspersed rounded lucent areas).
- ***Sepsis*** was identified as proven with at least one positive blood culture or other sterile site or was classified as suspected with negative blood cultures but clinical signs and biomarkers of septicemia.
- ***Seizure*** was recorded when a clinical manifestation of seizure was observed, requiring treatment with an antiepileptic drug.
- ***Necrotizing enterocolitis*** (NEC) was identified using at least one clinical criterion (bilious gastric aspirate or emesis, or abdominal distention, or occult/gross blood in stool without fissure) and at least one radiographic criterion (pneumatosis intestinalis, or hepato-biliary gas, or pneumoperitoneum). Classification of NEC stage was based on Bell Staging criteria (Hintz 2005)
- ***Intraventricular Hemorrhage*** (IVH) classification was based on Papile and Volpe's classification.
- ***Retinopathy of Prematurity*** (ROP) was assessed formally by an ophthalmologist among infants whose birth weight was ≤2000 gram or gestational age was ≤34 weeks.
- For hearing function, otoacoustic emission testing was carried out on 167/184 (91%) infants.

5. Follow-up schedule

Caregivers were provided with transportation if needed and were granted a waiver of payment when the cost was considered unaffordable. The family received a reminder phone call one week prior to each visit. For any drop-out, a reason was listed if known.

6. Data management

All data were encoded and entered in Excel files by the PI after each follow-up visit. After completing data collection, all data were re-entered by the PI. In case of differences, the original data from the paper case report forms were checked.

7. Data analysis

The Student's t-test was used for comparison of Bayley-III scores between preterm Vietnamese infants and healthy Vietnamese infants. The normal distribution of the scores was checked preferably by visualization, or by tests in R (e.g. Shapiro-Wilk test). The difference in the scores' variance was also checked before conducting the t-test.

8. Bayley Scales of Infant and Toddler Development-3rd Edition

The Bayley-III is an instrument administered to evaluate the developmental functioning of children from 1 to 42 months of age and to detect developmental delays. It is age-standardized and is one of the most commonly used tools in research and clinical settings, especially for assessing neurodevelopmental outcomes of preterm infants. The Bayley-III scores of healthy Vietnamese infants were provided for use in this study through a collaboration between the Oxford University Clinical Research Unit (OUCRU) and the research department of Children's Hospital 1.

9. Data on healthy Vietnamese infants

The Bayley-III data for healthy Vietnamese children were collected from a control cohort enrolled as part of a separate study evaluating enterovirus 71 infection and neurological development (ClinicalTrials.gov with the identification reference: NCT02066714). These children were recruited from kindergartens in District 8 in Ho Chi Minh City (HCMC), from a long-term birth cohort run as a collaboration between OUCRU and Hung Vuong Government Maternity Hospital and from three government primary care clinics, administered by the Preventive Medicine Centre in District 8, HCMC. The inclusion criteria for the healthy controls were as follows: age less than 4 years, no history of severe illness (cardiac, epilepsy, HIV), no intensive care admission, and no known developmental delay. A total of 267 children aged 3-43 months were enrolled and some were tested twice in 6 months (total of 476 assessments). Recruitment occurred between 2013-2014.

Table A. Neurodevelopmental scores of preterm Vietnamese infants at two years corrected age by gestational age.

Neurodevelopmental domains	Neurodevelopmental scores, mean (SD) or counts in number (%)		
	Gestational Age (GA)		Total
	GA < 32 weeks	GA ≥ 32 weeks	
	(n = 86)	*(n = 98)*	*(n = 184)*
Cognitive composite score, mean (SD), *n*	84.8 (8.4), *86*	84.2 (8.8), *98*	84.5 (8.6), *184*
Language composite score, mean (SD), *n*	90.5 (12.0), *84*	87.1 (12.8), *95*	88.7 (12.5), *179*
Motor composite score, mean (SD), *n*	93.1 (8.7), *86*	93.2 (9.3), *94*	93.1 (9.0), *180*
Cognitive subtest, mean (SD)	7.0 (1.7)	6.8 (1.8)	6.9 (1.7)
Receptive language subtest, mean (SD)	7.9 (1.9)	7.6 (2.1)	7.7 (2.0)
Expressive language subtest, mean (SD)	8.8 (2.6)	8.0 (2.7)	8.3 (2.7)
Fine motor subtest, mean (SD)	8.7 (1.8)	8.5 (2.1)	8.6 (1.9)
Gross motor subtest, mean (SD)	8.9 (1.7)	9.1 (2.0)	9.0 (1.9)
Cognitive composite score, n (%)	*n = 86*	*n = 98*	*n = 184*
< -2 SDs	17 (20)	15 (15)	32 (17)
[-2 SDs, -1 SD)	17 (20)	28 (29)	45 (24)
≥ -1 SD	52 (60)	55 (56)	107 (58)
Language composite score, n (%)	*n = 84*	*n = 95*	*n = 179*
< -2 SDs	4 (5)	10 (11)	14 (8)
[-2 SDs, -1 SD)	20 (24)	22 (23)	42 (23)
≥ -1 SD	60 (71)	63 (66)	123 (69)
Receptive Language subtests, n (%)	*n = 84*	*n = 95*	*n = 179*
< -2 SDs	4 (5)	9 (9)	13 (7)
[-2 SDs, -1 SD)	11 (13)	13 (14)	24 (13)
≥ -1 SD	69 (82)	73 (77)	142 (79)
Expressive Language subtests, n (%)	*n = 84*	*n = 95*	*n = 179*
< -2 SDs	13 (15)	20 (21)	33 (18)
[-2 SDs, -1 SD)	12 (14)	15 (16)	27 (15)
≥ -1 SD	59 (70)	60 (63)	119 (66)
Motor composite score, n (%)	*n = 86*	*n = 94*	*n = 180*
< -2 SDs	4 (5)	4 (4)	8 (4)
[-2 SDs, -1 SD)	12 (14)	11 (12)	23 (13)
≥ -1 SD	70 (81)	79 (84)	149 (83)
Fine Motor subtests, n (%)	*n = 86*	*n = 97*	*n = 183*
< -2 SDs	2 (2)	9 (9)	11 (6)
[-2 SDs, -1 SD)	18 (21)	17 (17)	35 (19)
≥ -1 SD	66 (77)	72 (74)	138 (75)
Gross Motor subtests, n (%)	*n = 86*	*n = 94*	*n = 180*
< -2 SDs	0 (0)	3 (3)	3 (2)
[-2 SDs, -1 SD)	16 (19)	9 (10)	25 (14)
≥ -1 SD	70 (81)	82 (87)	152 (84)

Table B. Descriptive statistics for candidate covariates and the outcome of interest (any NDI).						
No	Variable	Level	Number of infants	Any neurodevelopmental impairment		p-value
				No<br>n (%)	Yes<br>n (%)	
			184	83 (45.1%)	101 (54.9%)	
1	Gender	Boy	117	48 (41%)	69 (59%)	0.19
		Girl	67	35 (52%)	32 (48%)	
2	Gestational age	EVP	86	40 (47%)	46 (53%)	0.83
		MLP	98	43 (44%)	55 (56%)	
3	Multiple birth	1	158	71 (45%)	87 (55%)	0.99
		2	26	12 (46%)	14 (54%)	
4	Sepsis	No	59	28 (47%)	31 (53%)	0.85
		Yes	123	55 (45%)	68 (55%)	
5	Mechanical Ventilation	No	91	41 (45%)	50 (55%)	0.99
		Yes	92	42 (46%)	50 (54%)	
6	Surgery	No	150	68 (45%)	82 (55%)	0.99
		Yes	33	15 (45%)	18 (55%)	
7	Chronic lung disease	No	148	69 (47%)	79 (53%)	0.70
		Yes	34	14 (41%)	20 (59%)	
8	Maternal Education	Low	58	22 (38%)	36 (62%)	0.05
		Moderate	104	46 (44%)	58 (56%)	
		High	22	15 (68%)	7 (32%)	

Table C. Univariable regression analysis for the outcome of interest (any NDI)					
No	Variable	Level	Estimate	OR (95% CI)	p-value
1	Gender	Boy	Ref		
		Girl	- 0.45	0.64 (0.35 – 1.16)	0.14
2	Gestational age	1 week	0.04	1.04 (0.93 – 1.72)	0.48
3	Multiple birth	1	Ref		
		2	- 0.05	0.95 (0.41 – 2.19)	0.91
4	Sepsis	No	Ref		
		Yes	0.11	1.12 (0.6 – 2.08)	0.73
5	Mechanical Ventilation	No	Ref		
		Yes	- 0.02	0.98 (0.55 – 1.75)	0.94
6	Surgery	No	Ref		
		Yes	- 0.005	1.00 (0.47 – 2.12)	0.99
7	Chronic lung disease	No	Ref		
		Yes	0.22	1.25 (0.59 – 2.66)	0.57
8	Maternal age	1 year	- 0.03	0.97 (0.92 – 1.02)	0.18
9	Maternal Education	Low	Ref		
		Moderate	-0.26	0.77 (0.40 – 1.49)	0.44
		High	-1.25	0.29 (0.10 – 0.81)	0.02
